# Reprogramming of Retrotransposon Activity during Speciation of the Genus *Citrus*

**DOI:** 10.1093/gbe/evz246

**Published:** 2019-11-09

**Authors:** Carles Borredá, Estela Pérez-Román, Victoria Ibanez, Javier Terol, Manuel Talon

**Affiliations:** Centro de Genómica, Instituto Valenciano de Investigaciones Agrarias (IVIA), Valencia, Spain

**Keywords:** genomic evolution, insertion time, LTR retrotransposon, speciation, structural variations

## Abstract

Speciation of the genus *Citrus* from a common ancestor has recently been established to begin ∼8 Ma during the late Miocene, a period of major climatic alterations. Here, we report the changes in activity of *Citrus* LTR retrotransposons during the process of diversification that gave rise to the current *Citrus* species. To reach this goal, we analyzed four pure species that diverged early during *Citrus* speciation, three recent admixtures derived from those species and an outgroup of the *Citrus* clade. More than 30,000 retrotransposons were grouped in ten linages. Estimations of LTR insertion times revealed that retrotransposon activity followed a species-specific pattern of change that could be ascribed to one of three different models. In some genomes, the expected pattern of gradual transposon accumulation was suddenly arrested during the radiation of the ancestor that gave birth to the current *Citrus* species. The individualized analyses of retrotransposon lineages showed that in each and every species studied, not all lineages follow the general pattern of the species itself. For instance, in most of the genomes, the retrotransposon activity of elements from the SIRE lineage reached its highest level just before *Citrus* speciation, while for Retrofit elements, it has been steadily growing. Based on these observations, we propose that C*itrus* retrotransposons may respond to stressful conditions driving speciation as a part of the genetic response involved in adaptation. This proposal implies that the evolving conditions of each species interact with the internal regulatory mechanisms of the genome controlling the proliferation of mobile elements.

## Introduction

LTR retrotransposons are widespread mobile DNA detected in virtually every genome studied to date (Bao et al. 2015). They are found in great numbers due to their ability to replicate, as a new copy of each element is generated after a transposition event. It is well known that in their transposition mechanism three main motifs are involved (a reverse transcriptase, an RNase H, and an integrase, abbreviated RT, RH, and IN), whose order has been recurrently used to classify LTR retrotransposons in two main groups: *Copia* and *Gypsy* (Boeke and Corces 1989). Flanking the complete retrotransposon, two target site duplications (TSDs) produced by the element insertion are also found.

LTR retrotransposons are named after the two long terminal repeats flanking the element core that are identical upon insertion. Subsequently, each LTR accumulates mutations independently, an aspect that has been often used to date retrotransposon insertions (Pereira 2004; Hu et al. 2011; Xu and Du 2014; Liu et al. 2019). The homology between the LTRs of a single element also constitutes one of the main actors during the element excision that generally involves recombination. Unequal recombination (UR) between homologous LTRs from the same element leaves a single LTR surrounded by TSDs (soloLTR) (Devos et al. 2002). In contrast, when UR occurs between LTRs of different retrotransposons, one of the possible outcomes is a single LTR without flanking TSDs (Devos et al. 2002). Similarly, illegitimate recombination (IR) between nonhomologous elements is also relevant during retrotransposon purge, as it produces, among others, truncated elements with a single LTR and no TSDs (Devos et al. 2002; Vitte and Bennetzen 2006). LTRs produced by this mechanism are unpaired, but their formation mechanism is different from that of true soloLTRs; to differentiate both types of unpaired LTRs in this work, we will refer to LTRs produced by IR as nonsoloLTRs. Furthermore, the ratios between paired LTRs and soloLTRs have also been used to estimate retrotransposon purge rates in multiple studies (Vitte et al. 2007; Hawkins et al. 2009; Yin et al. 2015; Lyu et al. 2018).

Since their discovery, retrotransposons have proved their relevance in genome evolution, especially in repeat-rich plant genomes (Sanmiguel and Bennetzen 1998; Bousios et al. 2012). The effect of retrotransposons in plant evolution has been already described (Brookfield 2005; Du et al. 2009; Hanada et al. 2009; Sela et al. 2010; Butelli et al. 2012) highlighting their importance in adaptive processes (Vicient and Casacuberta 2017). Changes in retrotransposon activity have also been reported after drastic genomic events such as hybridization (Paz et al. 2015) and polyploidization (Parisod et al. 2009; Bardil et al. 2015; Mhiri et al. 2019) under the hypothesis of genomic shock (McClintock 1984), although other authors have found evidences against it (Göbel et al. 2018). It is also well accepted that environmental stresses may induce transposition, as well as the expression of genes neighboring residing transposons (Beguiristain et al. 2001; Kimura et al. 2001; Butelli et al. 2012; Dubin et al. 2018). The above premises strongly suggest that LTR retrotransposons might play a role in the evolutionary processes giving birth to distinct species. Associations between LTR retrotransposon activity and speciation have been certainly reported in rice and wheat (Mascagni et al. 2017; Zhang and Gao 2017), providing first insights on these connections. However, the recent establishment of solid phylogenies in several plant genera, such as in *Citrus* for instance (Wu et al. 2014, 2018), may allow these relationships to be explored in detail. Actually, retrotransposon activity in *Citrus* is a matter of increasing interest (Rico-Cabanas and Martínez-Izquierdo 2007; Du et al. 2018; Liu et al. 2019). The first retrotransposons found in *Citrus* were the *Copia*-like elements of sweet orange (Tao et al. 2005). Subsequent reports showed an enhancement on the CLCoy1 transposon activity under stress conditions in *Citrus limon* (De Felice 2009). Later, the expression of the Ruby gene, a major actor of the anthocyanin accumulation in blood oranges, was found to be regulated by a transposon promoter (Butelli et al. 2012, 2017). It has also been reported that the Mutator-like DNA transposon CitMule1 is responsible of the rearrangement of large genomic fragments in the genome of Clementine mandarin and therefore a major source of new Clementine genotypes and hence of new commercial varieties (Terol et al. 2015).

Although most of these works have focused on either a single genome or a reduced number of mobile elements, the growing interest of *Citrus* retrotransposons have led to the recent publication of two genome-wide surveys describing the retrotransposon landscape in different *Citrus* species, setting the background for deeper analysis. In the first study, LTR retrotransposons of *Citrusclementina* were mined and their phylogeny and distribution over the genome was described (Du et al. 2018). Later, the mobilomes of six species corresponding to five *Citrus* genomes of reference (Ichang papeda, pummelo, citron, Clementine, and sweet orange) and a relatively close-related genome (Chinese box orange) were the subject of a study, mainly focused in the MITE landscape of each genome (Liu et al. 2019). The authors also analyzed the phylogeny of the LTR retrotransposons, reaching results complementing those presented in Du et al. (2018) and in addition, estimated their average insertion times and half-life across the six genomes.

In this study, we expand these previous insights investigating LTR retrotransposon activity of the genus *Citrus* from an evolutionary context. To this end, we have used all *Citrus* reference genomes available today, corresponding to the six genomes previously used in Liu et al. (2019) plus two additional genomes of recent accessibility. Thus, the analyses included four true *Citrus* species: *Citrusichangensis* (Ichang papeda), *Citrusmaxima* (pummelo), *Citrusmedica* (citron) (Wang et al. 2017), and *Citrusreticulata* (mandarin) (Wang et al. 2018), and three different admixtures *of C. maxima* and *C. reticulata*, namely, *C. clementina* (Clementine mandarin) (Wu et al. 2014), *Citrusunshiu* (Satsuma mandarin) (Shimizu et al. 2017), and *Citrussinensis* (sweet orange) (Xu et al. 2013) in addition to *Severinia buxifolia* (Chinese box orange) (Wang et al. 2017). Out of these eight genomes, four of them consisted of thousands of scaffolds generated directly from Illumina sequencing (citron, Ichang papeda, Chinese box orange, and mandarin). However, those of sweet orange, pummelo and Satsuma and Clementine mandarins are all resolved up to the pseudomolecule scale, including nine main scaffolds corresponding to the nine *Citrus* chromosomes.

*Citrus* taxonomy and phylogeny have been a matter of controversy during the last century due to an unusually high number of interspecific hybrids that hinders the identification of pure species and prevents the inference of a reliable phylogeny. *Citrus* pure species reproduce through sexual crosses between members of the same species and therefore are generally free of introgression events. In contrast, most commercial or domesticated *Citrus* are derived from interspecific crosses followed by successive backcrosses, producing in this way characteristic admixture patterns that contain genomic regions from different pure species (Wu et al. 2014). Furthermore, commercial varieties are in general clonally propagated via grafting, which have allowed the admixture patterns that were generated many generations ago to reach our time. Although there are no clear evidences on the origin of the first admixed genomes, there are records of sweet oranges (an admixture between pummelo and mandarin) dated 2,300 years ago (cited in Xu et al. 2013), which might situate the origin of the first *Citrus* admixtures in the last few thousand years.

Of particular relevance for our goals are the comparative genomic analyses presented in Wu et al. (2014, 2018), that allowed the discrimination of pure and admixed *Citrus* accessions and inferred the phylogeny, genealogy, and chronology of the *Citrus* speciation. According to Wu et al. (2018), the phylogenetic relationship between the pure species of *Citrus* included in the current work is as follows. The Chinese box orange (*S.buxifolia*), an outgroup of the *Citrus* clade, diverged from the *Citrus* group ∼13 Ma (Pfeil and Crisp 2008). The *Citrus* last common ancestor lived in continental Southeast Asia ∼8 Ma, during the Late Miocene. This was a period of major climate changes characterized by a global CO_2_ level decline (Holbourn et al. 2018) that brought about a worldwide cooling epoch resulting in extensive weakening of monsoons and aridity enhancement of the subtropical regions (Herbert et al. 2016). In Southeast Asia, this marked climate alteration caused major changes in biota including rapid radiations of various plant lineages (see references in Wu et al. 2018) including *Citrus*. Ichang papeda diverged at the very beginning of *Citrus* speciation and apparently migrated to Central China. Shortly thereafter, two main clades separated ∼7–6 Ma: citrons and pummelos (India, Indochina, and the Malay Archipelago) in one of them and mandarins (East and South China and Japan) in the other. The three *Citrus* admixtures of *C. maxima* and *C. reticulata* studied here harbor different proportions of pummelo introgression in the mandarin genome (*C. clementina*, 12%; *C. unshiu*, 24%; and *C. sinensis*, 42%) and were generated at different historic times, at most few thousand years ago, from different genetic backgrounds.

Since variations in retrotransposon activity have been repeatedly related to environmental stresses in multiple plants, we found very tempting to analyze their fluctuations during *Citrus* speciation, a process most likely stimulated by a dramatic climate change, to elucidate if those environmental changes left any recognizable signature or imprint in their genomes. Thus, the goal of this study was first to describe the LTR retrotransposon landscape of the genus *Citrus* and then report the changes in their pattern of accumulation during the process of diversification that gave rise to the current *Citrus* species.

## Materials and Methods

### Genomic Data

All the genomic data were retrieved from public repositories. Eight reference genomes were used: four true pure *Citrus* species including *C.reticulata* (wild mandarin), *C.ichangensis* (Ichang papeda), *C.maxima* (pummelo), and *C.medica* (citron), two admixed (*C.reticulata*×*C.maxima*) commercial mandarins (*C.clementina* and *C.unshiu*, Clementine and Satsuma mandarins, respectively), one admixed (*C.* *maxima*×*C.reticulata*) commercial sweet orange (*C.sinensis*) and a close relative to the *Citrus* clade, *S.buxifolia* (Chinese box orange).

The reference genomes and the gene annotation data of *S. buxifolia*, *C. reticulata*, *C. maxima*, *C. medica*, *C. sinensis*, and *C. ichangensis* were downloaded from http://citrus.hzau.edu.cn/;last accessed November 21, 2018. The *C. unshiu* genome and annotation data were downloaded from http://www.citrusgenome.jp/;last accessed November 21, 2018. The *C. clementina* reference genome and its annotation data were downloaded from Phytozome (*Citrus clementina* v1.0).

Paired-end Illumina reads for the structural variant analysis were retrieved from the NCBI Sequence Read Archive. The codes and equivalence of each accession are available in the supplementary table 1, Supplementary Material online.

### Detection and Classification of LTR Retrotransposon Cores

Putative LTR retrotransposons were found and validated in *C. clementina* reference genome using an integrated detection pipeline, LocaTR (Mason et al. 2016), which combines the results from several LTR retrotransposon detection tools (McCarthy and McDonald 2003; Sperber et al. 2007; Ellinghaus et al. 2008). Results from LTR_FINDER (Xu and Wang 2007) were also incorporated following the user manual of LocaTR to generate a comprehensive set of LTR retrotransposons.

A curated retrotransposon database, Gypsy Database (Llorens et al. 2011), was searched to retrieve protein and DNA sequences of three LTR retrotransposon domains (IN, RT, and RH) of every GyDB element annotated. To retrieve DNA sequences from the core retrotransposon domains, BlastX analyses were performed using as queries each of the *C. clementina* and GyDB retrotransposon DNA sequences against a custom GyDB core domain protein sequences. Only hits with an e-value below 1×10^−20^ and containing the three core domains (IN + RT + RH, regardless of the order) in the *C. clementina* putative retrotransposons were selected. Each *C. clementina* element was classified as *Gypsy* or *Copia* depending on the order of their domains: RT–RH–IN as *Gypsy* and IN–RT–RH as *Copia*.

The *C. clementina* retrotransposon core collection was used as query in a BlastN analysis against eight reference genomes: *C. clementina*, *C. ichangensis*, *C. reticulata*, *C. unshiu*, *C. maxima*, *C. medica*, *C. sinensis*, and *S. buxifolia*. Only hits covering over 80% of the query and with an e-value lower than 1×10^−25^ were selected, and overlapping hits were merged. Hits produced by *Copia C. clementina* elements were classified as belonging to the *Copia* superfamily, and the same was done with the *Gypsy* superfamily.

Retrotransposon cores sharing over 80% of sequence identity in at least 80% of the sequence length, with a minimum of 80 bp covered were independently clustered in each genome using a modified mean shift algorithm implemented in MeShClust (James et al. 2018), and each cluster was assigned to a new retrotransposon family following the system of Wicker et al. (2007). The longest sequence of each family was selected as a cluster representative. Family representatives from *Copia* and *Gypsy* superfamilies were aligned with a GyDB prealigned profile. Both alignments were performed using MAFFT L-INS-I algorithm (Katoh and Standley 2013). A maximum likelihood phylogenetic tree was built with FastTree (Price et al. 2010) and the tree topology was explored using R and ggtree (Yu et al. 2017; R Core Team 2018).

### *Citrus* LTR and Retrotransposon Distribution

Each reference genome was split in nonoverlapping windows of up to 1 Mb and each retrotransposon was associated to one of them, together with the gene content of each window. For scaffolds >100 kb but <1 Mb, the complete scaffold was used as a single window. Scaffolds <100 kb were discarded. The median genic content among the windows of *C.* *clementina* was estimated and used to roughly locate the pericentromeric regions.

Although the LocaTR pipeline is capable of detecting large amounts of LTR retrotransposons, it does not separately annotate LTRs. One of the tools integrated in LocaTR, LTR_Harvest, was used to detect paired LTRs. To do so, each LTR retrotransposon core and 30 kb of flanking sequences were used as queries for LTR_Harvest. The representativity of the new LTR_Harvest data set of the original data set found by homology search was manually verified by checking if the proportions of retrotransposons found in each lineage and species are roughly conserved across the two data sets (supplementary fig. 1, Supplementary Material online). As every LTR defined by LTR_Harvest must have a pair, the two LTRs of each LTR_Harvest detected element were aligned using MAFFT (Katoh and Standley 2013), and the Kimura-2-parameters distance was assessed for each alignment using DiStats (Astrin et al. 2016). The conversion of Kimura-2-parameters distance to time was calculated using as mutation rate 4×10^−9^ and 5×10^−9^ substitutions per year, as previously reported (De La Torre et al. 2017), multiplied by a factor of two as in Hu et al. (2011).

A BlastN search was used to find sequences similar to the paired LTRs identified by LTR_Harvest, selecting hits with an identity of over 80% across 90% of the query (hits closer than 100 bp were merged). For each hit, a dot plot was performed against 30 kb of their flanking sequence using YASS (one seed to consider a hit and an Xdrop threshold score of 100 were used, the remaining parameters were left as by default) (Noe and Kucherov 2005). Hits flanked with at least one similar (a hit extending over 90% of the sequence) copy of themselves were classified as paired LTRs. The remaining hits were considered unpaired LTRs (unpaired LTRs). Unpaired LTRs were then searched for TSDs to classify them in true solo-LTRs or nonsolo-LTRs. To do so, the 20 bp flanking both sides of each unpaired LTR were searched for identical kmers of lengths from four to seven nucleotides using inhouse scripts. If a kmer was found in the two 20-nucleotide flanking sequences, it was defined as a TSD and the unpaired LTR was classified as a solo-LTR. In any other case, the unpaired LTR was classified as a nonsolo-LTR. Every LTR regardless of its type was associated to position-based windows as in the case of genes and complete retrotransposon cores.

### Determination of Unpaired LTRs Closest Relatives

Each unpaired LTR (soloLTR or nonsoloLTR) was used as a query in a BlastN analysis against a database including all the LTRs found (paired and unpaired). The best hit for each sequence (excluding the sequence itself) was recorded provided it covered at least 90% of the query with 90% of identity. Only reciprocal best hits (A’s best hit is B and B’s best hit is A) were selected, and the reference genomes of the query sequence and the hit were recorded.

### Determination of Transposition Events via Structural Variant Detection

Illumina paired-end reads from 43 mandarin accessions (supplementary table 1, Supplementary Material online) were retrieved from SRA. Reads with over 30% of their bases showing a quality score <30 were discarded, and the remaining were aligned against the *C. clementina* reference genome using bwa-mem (Li 2013).

Structural variants were discovered using Lumpy 0.2.13 and SVTyper 0.1.3 (Layer et al. 2014; Chiang et al. 2015). Deletions with a size <100 kb and with a reciprocal coverage of 80% between them and any complete LTR retrotransposon found by LTR_Harvest (at least 80% of the deletion annotated as a retrotransposon and vice versa) were selected and assigned as retrotransposon-induced deletions. This process was independently applied to each sample. Deletions supported by at least 20% and 80% of the reads were considered hemizygous and homozygous, respectively.

### Statistical Analyses and Data Representation

Correlation tests were performed using the nonparametrical Spearman rank correlation test implemented in R stats package (v3.5.1). Phylogenetic trees were plotted using ape, ggplot, and ggtree (Wickham 2016; Yu et al. 2017; Paradis and Schliep 2018). The remaining plots were created using ggplot.

## Results

### LTR Retrotransposon Detection and Classification

Using a combined detection approach, 2,666 putative LTR retrotransposons were found in the *C.clementina* haploid reference genome. Of them, 2,376 contained exactly one copy of each of the three core motifs (integrase, RNAse H, and reverse transcriptase) of the LTR retrotransposons and were consequently annotated as LTR retrotransposons. These LTR retrotransposons were then used as queries to identify similar elements in eight reference genome sequences (*S.buxifolia*, *C.ichangensis*, *C.maxima*, *C.medica*, *C.reticulata*, *C.clementina*, *C.unshiu*, and *C.sinensis*), retrieving a total of 32,506 retrotransposon cores, which were classified in the *Gypsy* or *Copia* superfamilies depending on their motif order (table 1).

**Table 1:** ***Citrus* LTR Retrotransposon Elements and Families** ^a^

Superfamily	Lineages	*Citrus clementina*	*Citrus sinensis*	*Citrus unshiu*	*Citrus maxima*	*Citrus medica*	*Citrus ichangensis*	*Citrus reticulata*	*Severinia buxifolia*	Total
*Copia*	Tork	1,001 [87]	556 [69]	721 [65]	1,072 [86]	1,294 [74]	1,453 [110]	1,073 [59]	864 [74]	**8,034 [624]**
	SIRE	538 [15]	340 [12]	600 [9]	786 [18]	926 [16]	99 [27]	424 [19]	260 [9]	**3,973 [125]**
	Oryco	123 [17]	92 [18]	102 [14]	69 [22]	191 [17]	128 [22]	121 [18]	118 [23]	**944 [151]**
	Retrofit	483 [58]	685 [61]	429 [80]	497 [56]	227 [47]	581 [56]	495 [53]	284 [57]	**3,681 [468]**
	Total *Copia*	**2,145 [177]**	**1,673 [160]**	**1,852 [168]**	**2,424 [182]**	**2,638 [154]**	**2,261 [215]**	**2,113 [149]**	**1,526 [163]**	**16,632 [1,368]**
*Gypsy*	Tat	386 [36]	212 [33]	246 [27]	351 [51]	402 [21]	271 [31]	309 [34]	131 [29]	**2,308 [262]**
	Athila	1,510 [72]	830 [37]	768 [56]	1,974 [58]	1,355 [30]	886 [47]	1,068 [36]	608 [28]	**8,999 [364]**
	Galadriel	108 [21]	88 [21]	80 [28]	131 [26]	107 [20]	85 [23]	111 [19]	72 [26]	**782 [184]**
	Del	84 [23]	66 [13]	84 [24]	91 [25]	70 [15]	112 [20]	19 [15]	81 [17]	**607 [152]**
	CRM	209 [34]	148 [24]	205 [30]	336 [40]	213 [31]	268 [33]	203 [22]	234 [20]	**1,816 [234]**
	Reina	163 [42]	128 [53]	360 [48]	141 [64]	157 [47]	157 [51]	118 [41]	138 [64]	**1,362 [410]**
	Total *Gypsy*	**2,460 [228]**	**1,472 [181]**	**1,743 [213]**	**3,024 [264]**	**2,304 [164]**	**1,779 [205]**	**1,828 [167]**	**1,264 [184]**	**15,874 [1,606]**
Total LTR		**4,605 [405]**	**3,145 [341]**	**3,595 [381]**	**5,448 [446]**	**4,942 [318]**	**4,040 [420]**	**3,941 [316]**	**2,790 [347]**	**32,506 [2,974]**

a Family numbers are shown in brackets.

**Fig. 1.**
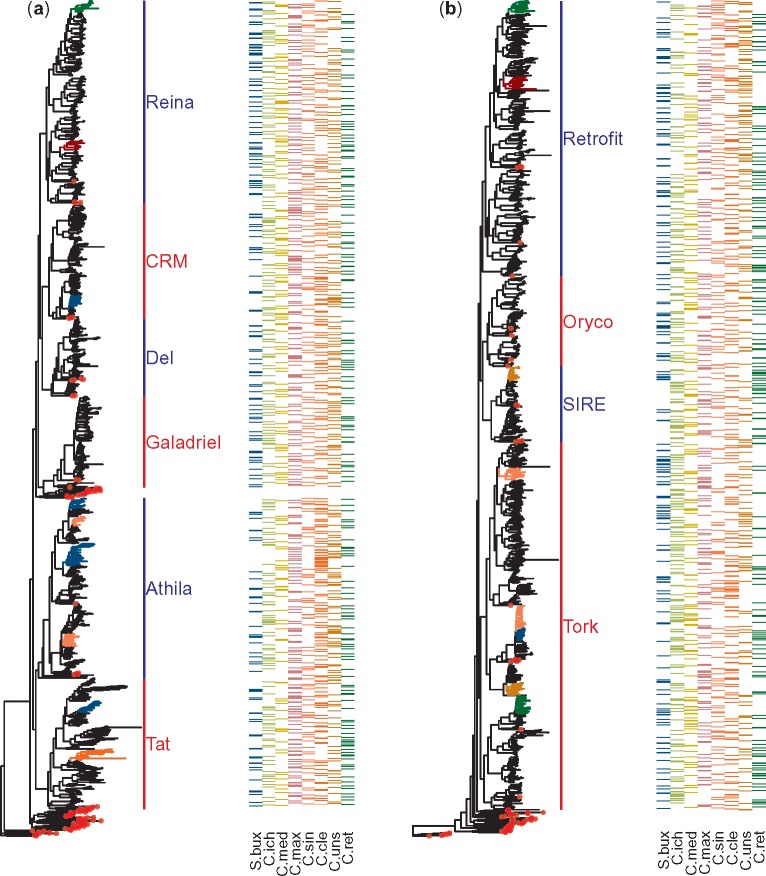
—*Citrus* LTR retrotransposon phylogenetic trees and presence across species. Phylogenetic trees of LTR retrotransposon families belonging to *Gypsy* (*a*) and *Copia* (*b*) superfamilies are shown. Next to each tree a heatmap indicate the species of origin for each family (terminal node). Red dots mark terminal nodes belonging to sequences from the curated transposon database GyDB. Colored branches represent clades with over 20 terminal nodes not harboring families from the eight references studied. The color legend is the same as that of the heatmap, with clades missing two or more references highlighted in dark red. The following naming convention is used to refer to the reference genomes: S.bux, *Severinia buxifolia*; C.ret, *Citrus reticulata*; C.ich, *Citrus ichangensis*; C.max, *Citrus maxima*; C.med, *Citrus medica*; C.sin, *Citrus sinensis*; C.uns, *Citrus unshiu*; C.cle, *Citrus clementina*.

All cores within each genome were grouped in families. The number of LTR retrotransposon families detected among the eight genomes varied between 316 and 446, accounting for 2,974 families in total (table 1). The longest sequence of each family was aligned with a representative set of sequences from GyDB and two independent phylogenetic trees were built for *Gypsy* (fig. 1*a*) and *Copia* (fig. 1*b*) retrotransposons. Every *Citrus* retrotransposon family was classified in one of the following plant retrotransposon lineages: Retrofit, Oryco, SIRE, or Tork lineages for *Copia* retrotransposons, and CRM, Reina, Del, Galadriel, Athila, or Tat lineages for *Gypsy* retrotransposons.

To study the de novo acquisition and loss of retrotransposon families the topology of each phylogenetic tree was explored. As retrotransposon families were independently defined in each genome, those shared by several genomes are clustered together in the phylogenetic tree as a clade containing multiple nodes, and with at least one member per genome. In contrast, family gains and losses are defined by clades whose families were present in many but not all the genomes. All clades harboring >20 terminal nodes were analyzed, and those missing one or more reference genomes among their nodes were identified (fig. 1). Although most of the 20-node clades comprise a sequence from each reference genome, a small number of clades (8 in *Copia* and 9 in *Gypsy* trees) harbored families missing in some species. Out of these 17 clades, 5 of them were missing a representative in the reference genome of *S. buxifolia*, the most distant genome included in this work.

### Accumulation Patterns and Dating of Complete LTR Retrotransposons

**Fig. 2.**
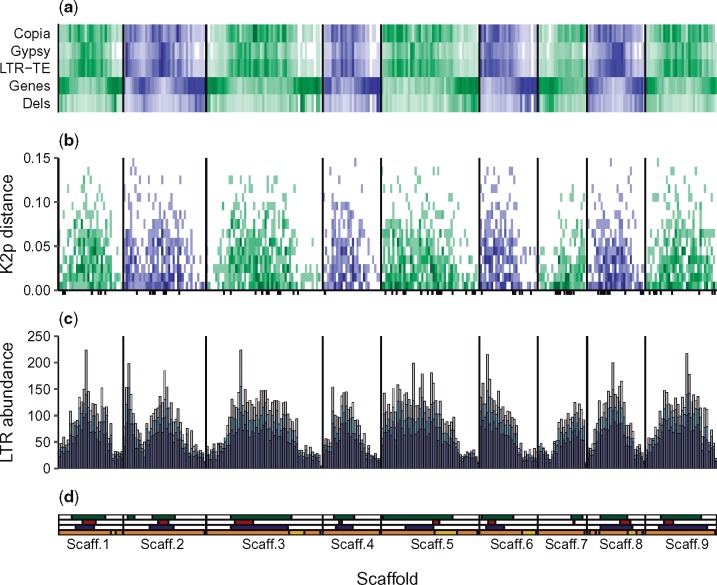
—LTR retrotransposon abundance, age, and activity in the Clementine reference genome. Only the nine main scaffolds of the Clementine reference are shown. All results are summarized in 1 Mb windows. (*a*) Distribution of LTR retrotransposons (LTR-TE) disaggregated into *Copia*, *Gypsy*, and total elements. Below, the per Mb genic content is shown. On the lowermost row, a per-window average of the transposon-associated deletions across 43 mandarin genomes is shown, the full data can be found in supplementary figure 2, Supplementary Material online. The intensity of each bin is proportional to the percentage of bases covered per window, with the maximum intensity normalized to the maximum value in each row. (*b*) LTR-based dating retrotransposons in *Citrus clementina*. The relative age was calculated as the Kimura-2-parameters genetic distance (K2p) (Hu et al. 2011) between LTR pairs. Each LTR retrotransposon was classified in an age interval (windows of 0.01 distance units) and genomic position. The coordinates of each bin are given by the genomic position of each element and its age, and the intensity is proportional to the number of transposons included in the bin. Elements with identical LTRs (K2p distance equals 0) are marked as black ticks under the *x* axis. (*c*) Total number of soloLTR (purple), nonsoloLTR (blue), and pairedLTRs (gray) across the *C. clementina* reference genome, shown as a stacked bar plot. Total LTR (totalLTRs) counts are given by the total height of each bar. (*d*) Genomic features of the *C. clementina* reference genome. On top, the centromeres predicted in this work based on the genic content (green), together to those of Aleza et al. (2015) (red) and (Wu et al. 2014) (blue). The last row shows the admixture map of the *C. clementina* haploid reference genome: genomic fragments coming from mandarin and pummelo are shown in orange and yellow, respectively, while fragments with unknown precedence are shown in gray. The data were obtained as explained in Wu et al. (2014).

The genomic position of each LTR retrotransposon core of the *C. clementina* reference was used to study the retrotransposon core accumulation patterns along the genome. When the distribution of the LTR retrotransposon cores of *C. clementina* was studied (fig. 2*a*), a negative correlation between gene content and LTR retrotransposon abundance was found (*p*-value <0.05). This association was also independently observed for each genome (supplementary table 2, Supplementary Material online). In contrast, retrotransposon activity hotspots, characterized by a higher frequency of retrotransposon-induced deletions, were mostly found in genic regions of *C. clementina* (fig. 2*a*), as further discussed in subsequent sections of this work.

Paired LTRs were found flanking 3,102 out of the 4,605 similarity-found retrotransposon cores in Clementine, allowing for the determination of complete elements, with an average length of 8,701 bp. Considering the eight genomes, a total of 18,630 complete retrotransposons with a global average of 8,208 bp in length were detected (table 2). The average genome proportion of LTR retrotransposons was calculated per species considering in each case the species average element length, the number of elements and the total genome length. These proportions ranged from 3.60% to 9.97% among the different species but are most probably an underestimation of the real values, as they are solely based on full-length LTR retrotransposons with well-defined LTRs, disregarding a considerable amount of retroelements. By considering each retrotransposon core as part of a complete element, the maximum LTR retrotransposon content was calculated per species (assigning to each core the genome-specific average length), which yielded a retrotransposon proportion ranging from 6.87% to 15.93% in the eight genomes studied (table 2).

**Table 2:** *Citrus* LTR Retrotransposon Length, Number, and Coverage

Organism	LTR-TE Length and Number		Genome coverage (%)		
	Cores Length and Number^a^	Complete Elements Length and Number^a^	LTR-TE Cores	Complete LTR-TE	Max. LTR-TE^b^
*Citrus clementina*	2,650 [4,605]	8,701 [3,102]	4.00	8.84	13.13
*Citrus sinensis*	2,469 [3,145]	7,860 [1,531]	3.20	4.95	10.17
*Citrus unshiu*	2,564 [3,595]	8,097 [1,777]	2.53	3.95	7.99
*Citrus maxima*	2,627 [5,448]	8,940 [3,410]	4.68	9.97	15.93
*Citrus medica*	2,600 [4,942]	8,137 [2,863]	3.16	5.73	9.89
*Citrus ichangensis*	2,595 [4,040]	8,057 [2,357]	2.93	5.31	9.10
*Citrus reticulata*	2,587 [3,941]	8,087 [2,129]	2.95	4.97	9.21
*Severinia buxifolia*	2,563 [2,790]	7,792 [1,461]	2.26	3.60	6.87
All species	2,590 [32,506]	8,308 [18,630]	3.18	5.85	10.21

a Number of elements is shown in brackets.

b Considering the total core number and the complete element length.

The genetic distance between both paired LTRs of each element was then used to estimate its insertion time (Hu et al. 2011). The oldest LTR retrotransposons were generally found in pericentromeric regions where they were visibly more abundant, although this differential distribution was progressively less evident as younger elements were considered (fig. 2*b*). Elements containing two identical LTRs (distance equals 0) have been previously defined as newly inserted elements (Xu and Du 2014). In *C.clementina*, 87 of these new elements were found all across the genome in a distribution which was not dependent on the genic content (supplementary table 2, Supplementary Material online and fig. 2*b*), which might indicate an unbiased insertion along the genome for the most recent *C. clementina* retrotransposons. Retrotransposon insertion times were then calculated for each species, and the same lack of correlation was observed when all species were considered except in the case of *C. maxima* and *C. sinensis*, in which new LTR retrotransposons were significantly less common in genic regions possibly indicating a biased insertion (supplementary table 2, Supplementary Material online).

**Fig. 3.**
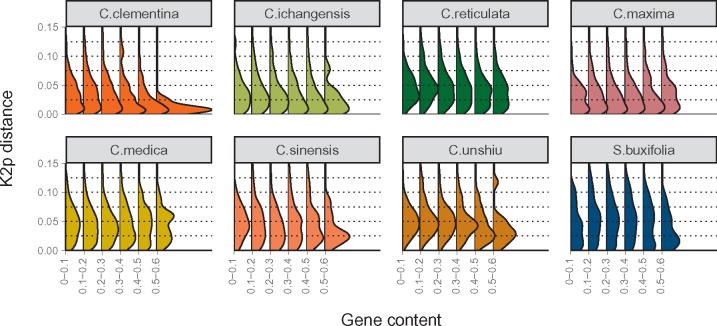
—Relative age distribution of paired LTRs per species and gene density. Panels show the eight reference genomes and contain six retrotransposon age distributions each, one per genic-content bin. In each distribution, the height of the curve represents has been normalized to represent the proportion of elements with a given pairwise distance between their LTRs.

Genomes were divided in windows of 1 Mb that were assigned to one of six categories regarding their gene content (from 0% to 60% of the window covered by genes, in 10% bins). Each retrotransposon was assigned to one genomic region based on their position in the genome, and the age distribution per gene-content bin and per species was calculated (fig. 3). Among all the studied genomes, the correlation between the genic content and the LTR retrotransposon age distribution was not consistent. In *C. clementina*, young elements were present along the genome regardless of the gene content, while older elements became progressively less common as the genic content dropped. This results in an age distribution with an abundance peak becoming more prominent as the genic content increases (fig. 3). Similar but less pronounced patterns were also found in *C. ichangensis*, *C. sinensis, C. reticulata*, and *C. unshiu*. On the other hand, *C. maxima* and *C. medica* showed a more uniform age distribution across different gene content levels. Finally, *S. buxifolia* followed a different distribution, without visible changes except for the last category (comprising the highest gene density) that reveals a very recent accumulation of young elements in genic regions.

Moreover, the purge rate of LTR retrotransposons in *C.* *clementina* was determined studying the proportion of soloLTR, nonsoloLTR, and paired LTR across the genome (fig. 2*c*). Based on these proportions, we conclude that the retrotransposon elimination in *C.clementina* occurs at a faster rate in genic regions (see below).

Finally, the location of pericentromeric regions in the *C. clementina* genome was calculated. The overall median genic content across the whole *C. clementina* genome was determined to be 23%. Up to ten 1-Mb windows were assigned as pericentromeric regions along the nine main scaffolds as their genic content fell below that threshold (fig. 2*d*). Consistently, the centromere locations correlated with retrotransposon abundance, their aging, and the presence of activity hotspots.

### Retrotransposon Activity Patterns among Mandarins

An indicator of retrotransposon recent activity in resequenced genomes is the presence of retrotransposon-induced deletions that are easily evidenced after comparison with the reference genome. Deletions could be generated by either a true deletion of the element in the resequenced accession via one of the methods mentioned above, or through an insertion of that element in the reference genome after its divergence from the resequenced genome (Rahman et al. 2015).

In principle, the strategy followed in this work could certainly detect novel element insertions since it is expected that these elements would be completely missing in the resequenced genome. For retroelement true deletions, the observed deletion would span across most of the retrotransposon, except for the LTRs that consequently remain in both, the resequenced and the reference genomes. Unfortunately, reads mapped within a retrotransposon (such as those that would support these deletions) are usually unreliable due to the repetitive nature of mobile elements. For this reason, deletions reciprocally spanning over 80% of an element (see Materials and Methods) were assigned as either insertions or deletions, without distinguishing between them.

The distribution of retrotransposon-induced deletions across 43 mandarin accessions (supplementary table 1, Supplementary Material online) was studied to identify retrotransposon activity hotspots across the Clementine genome. A total of 15,388 deletions spanning over LTR retrotransposons were annotated (see Materials and Methods) with an average of 358 deletions per sample, all of them ranging from 2,515 to 15,378 bp (the average length was 7,818 bp). Their genomic coordinates were used to study the retrotransposon activity across the genome, which was significantly higher in genic regions (fig. 2*a* and supplementary fig. 2, Supplementary Material online).

### Cross-Homology of Unpaired LTRs among *Citrus*

**Fig. 4.**
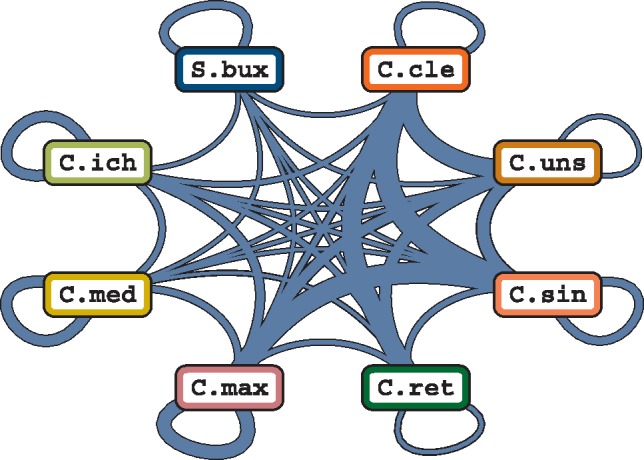
—Unpaired LTR-relatedness network. The width of the line between every pair of species is proportional to the number of shared soloLTRs and nonsoloLTRs. Loops indicate elements whose closest relative is found in the same genome. Only reciprocal hits were considered, and hence, no directionality is required. The same naming convention as that of figure 1 is used.

Each unpaired LTR was queried against the total LTR collection to find its closest relative, and the genome harboring it was recorded in each case (fig. 4). *Citrusclementina* unpaired LTR closest relatives were mostly found in *C. sinensis*, *C. reticulata*, and *C. unshiu*, all of them containing great amounts of mandarin genome as they are either mandarin admixtures (*C. sinensis*, *C. clementina*, and *C. unshiu*) or a pure mandarin itself (*C. reticulata*). The remaining Clementine unpaired LTR relatives were found mainly in the other pure species involved in Clementine’s admixture, *C. maxima*, followed by more distant *Citrus* species such as *C. ichangensis* and *C. medica*. A small proportion of the Clementine unpaired LTRs showed a significant homology to those of *S. buxifolia*. It is worth highlighting that *C. clementina* unpaired LTR have by definition their pairs excised and therefore the number of closely related unpaired LTR within the same genome should be lower than that of closely related admixtures, in which the generation of an unpaired LTR from the same retrotransposon has not taken place necessarily.

For the remaining admixtures, a similar pattern was found, in which the majority of unpaired LTR had their closest relatives in either other admixtures or the pure species that gave rise to them. In contrast, in the pure species *C. medica*, *C. ichangensis*, and *S. buxifolia*, most unpaired LTR found their closest relatives within the same genome, probably because they correspond to multiple insertions of similar elements. The case of *S. buxifolia* is especially remarkable, with 65% of its unpaired LTR having their closest relative within the same genome and only 35% of them being more similar to elements found in the *Citrus* genomes.

### Accumulation Patterns of Long-Terminal Repeats across the Genome

In the Clementine genome, a total of 31,221 LTRs (total LTR) were found by similarity with those detected by LTR_Harvest (fig. 2*c*). Of them, 9,826 were paired LTRs, that is, they have at least one similar LTR in their flanking 30 kb. Of the remaining unpaired LTRs, 15,471 were identified as true soloLTRs as they were flanked by a 4–7 bp long TSD. Finally, 5,924 LTRs were found unpaired and lacking any TSD signature, thus being marked as nonsoloLTRs probably produced by IR or interelement UR. The remaining four LTRs showed no homology with themselves, probably due to a misassignment as complete LTRs, and were discarded for further analysis. The pairedLTR: soloLTR: nonsoloLTR ratio was 1:1.57:0.60.

When the same methodology was applied to the set of species analyzed, a similar proportion of paired LTRs, soloLTRs, and nonsoloLTRs were found. In this case, 96,381 paired LTRs were detected. The number of soloLTR and nonsoloLTR was 123,743 and 54,009, respectively. 22 LTRs were discarded for the same reasons as above, and the final pairedLTR: soloLTR: nonsoloLTR ratio was 1:1.28:0.56.

By considering in a per-window basis the genic content, the number of paired, solo, and nonsolo LTR and their proportion related to the total number of LTRs, the correlation between purge rate and gene content was established (supplementary table 2, Supplementary Material online). A negative correlation between total LTRs and genes was found in all genomes. When genic content was compared with the proportion of soloLTRs over total LTRs, a positive correlation was detected, indicating that soloLTR are more common in gene-rich regions. In contrast, nonsoloLTRs showed a positive correlation with the genic content in *C. medica*, but also a negative correlation in *C. ichangensis* and *C. unshiu*. Finally, the proportion of paired LTRs, which should be a proxy of the complete retrotransposon abundance, was negatively correlated with the genic content in all but *C. ichangensis* genomes.

### Evolution of Retrotransposon Activity among *Citrus* Genomes

**Fig. 5.**
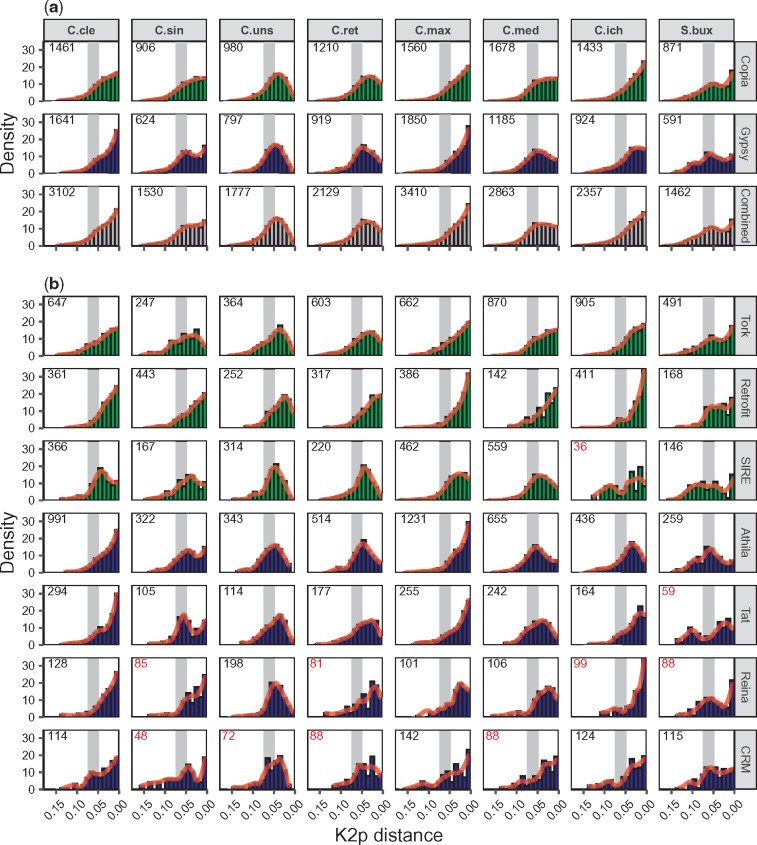
—Retrotransposon activity pattern per species and lineage. Retrotransposon activity evolution over time. For each species, retrotransposons were grouped either in (*a*) superfamilies or (*b*) lineages. The proportion of retrotransposons falling in each specific age bin is shown, the total transposon numbers per each species and superfamily or lineage is shown in the top left corner. Histograms containing <100 observations had this number in red. Members from *Gypsy* and *Copia* superfamilies are colored green and blue, respectively. In gray, the proposed date for the *Citrus* radiation giving rise to the species studied (7.5–6.0 Ma) converted to distance units (0.075–0.048 K2p units) (Hu et al. 2011) is shown. Species naming convention are as in figure 1.

The distribution of the number of LTR retrotransposons dated at a certain age was used as a proxy of the activity of elements belonging to a specific lineage or superfamily at that given age (fig. 5*a* and *b*).

The number of retrotransposons dated at each age evolved similarly over time within each genome in both *Copia* and *Gypsy* superfamilies. However, when different species were compared, this similitude was no longer observed (fig. 5*a*). In the leftmost part of each plot, representing the oldest retrotransposons, the number of elements steadily increased with the age following a gradual rise in all eight species. However, starting from 0.06 K2p distance units, this pattern was no longer maintained among species (fig. 5*a*). Instead, from this point the age distribution in each species followed one of three different models: a) in the case of *C. clementina*, *C. maxima*, and *C. ichangensis*, it increased progressively over time following an almost exponential pattern of growth; b) in *C. medica*, *C. reticulata*, and *C. unshiu*, it was first arrested and then reduced, either slightly or considerably; c) in *C. sinensis* and *S. buxifolia*, it followed a third pattern similar to the previous model (b) except for a final recent burst.

When LTR retrotransposon superfamilies were disaggregated into lineages, their differences became more noticeable. In each of the species analyzed, different retrotransposon lineages followed distinct patterns that often differed from the species-specific patterns (fig. 5*b*). In 32 out of 46 reliable histograms (those including at least 100 elements), the retrotransposon age distribution resembled that of the species (fig. 5*a*). In some cases, a general trend in all lineages on a single species (or vice versa) was found, but every time some exceptions arose. For example, all lineages on *C. maxima* and *C. clementina* genomes were exponentially growing, except for SIRE and Reina elements. Conversely, Retrofit elements seemed to grow exponentially in all species except in *C. unshiu*, *C. reticulata*, and *S. buxifolia*; meanwhile, SIRE element distribution peaked at some point in the past in every genome except in *Severinia*, and its activity started to decay since then.

## Discussion

### The Retrotransposon Landscape in *Citrus*

*Citrus* retrotransposons have recently seen a growing interest, especially since the publication of several reference genomes that have enabled high throughput retrotransposon surveys to be performed. The results presented above generally agree with two previous descriptive works reporting the retrotransposon landscape in different *Citrus* genomes (Du et al. 2018; Liu et al. 2019). We have found 32,506 retrotransposon cores in eight genomes, and approximately half of them were annotated as full-length elements since they were flanked by two LTRs (the presence of other retrotransposon features such as a polypurine tract or a primer binding site was not verified). The average length of these complete retroelements, calculated both from the LTR-Harvest results and from the retrotransposon-induced deletions in *C. clementina*, was slightly above 8 kb per LTR retrotransposon, a length roughly conserved in the eight reference genomes (table 2) and in agreement with the two abovementioned reports (Du et al. 2018; Liu et al. 2019). The average retrotransposon length was used to estimate the percentage of the genome covered by complete retrotransposons that ranged from 3% to 10% of the genome (table 2). These proportions were higher in the two better resolved genomes (*C. clementina* and *C. maxima*), possibly due to the difficulties in the detection of retrotransposons in Illumina-generated references. The retrotransposon abundances found for the different genomes largely agree with those of Clementine (Du et al. 2018) but are not in concordance with the results published by Liu et al. (2019), that reported values ∼30% in six of the eight genomes studied in this work. These discrepancies might arise due to an overestimation of the retrotransposon collection, especially if fragmented retrotransposons were taken into consideration. In general, big genomes tend to contain higher proportions of mobile elements than smaller ones, as observed in maize (>2 Gb genome size, 75% LTR retrotransposons) (Baucom et al. 2009) and Arabidopsis (160 Mb, 6%) (Pereira 2004), although rice for instance (390 Mb, 35%) (Sasaki 2005) exhibits an intermediate situation.

Retrotransposon cores were grouped in families that could be classified in ten plant retrotransposon lineages, as reported in *C. clementina* (Du et al. 2018). Our results are also comparable with those reported in Liu et al. (2019), even though the use of a different retrotransposon lineage nomenclature hinders a direct comparison, an issue already encountered by other authors (Neumann et al. 2019). Overall, the data show that only these ten retrotransposon lineages can be found across the multiple *Citrus* genomes. Interestingly, the great majority of the retrotransposon families of *Citrus* are present in all the genomes analyzed (fig. 1) and even in the distant species *S. buxifolia* that diverged from *Citrus* 13 Ma (Pfeil and Crisp 2008), suggesting that most retrotransposon families were already hosted by the common ancestor of both. We also identified 17 families that were absent in some species and among them, five were not detected in S. buxifolia. Failure to detect every member of a family of LTR retrotransposons in a species is unexpected to occur due to technical limitations because these families are in general composed of numerous members inserted in different genomic positions. The absence of a given family in a specific species might be the result of insertions or deletions of retroelements, such as the colonization of a specific genome after its divergence with the remaining species (Piednoël et al. 2013) or the depletion of a whole family previous to their proliferation, when the copy number remains low in the genome (Rahman et al. 2015). An alternative explanation for undetected retrotransposon families is the process of incomplete lineage sorting that can generate inconsistent genetic signals when alleles not fixed in a population are studied. Incomplete linage sorting has been considered in the field of plant phylogenetics (Strickler et al. 2015; Zhou et al. 2017) and has also been proposed as an explanation to unexpected retrotransposon presence/absence patterns in animals (Suh et al. 2015; Kuritzin et al. 2016; Doronina et al. 2017). Since only one sampled individual per species was analyzed in this work, we cannot reject the possibility that some of the missing clades are produced by this process. Finally, de novo acquisition of families via hybridization or horizontal transfer, events already described in plants, may also be considered (Roulin et al. 2009; El Baidouri et al. 2014). Although any of the above mechanisms may in principle cause the apparent loss of these 17 families, the 5 retrotransposon families missing *S. buxifolia* presumably colonized the *Citrus* genomes after their divergence with the genus *Severinia*.

We further investigated the relatedness between the retrotransposons present in the distinct species by estimating the degree of LTR sharing (fig. 4). In most pure species, the closest relative to each unpaired LTR was found in the same genome. This was expected, since retrotransposition events intrinsically generate copies of the same element and, before the first transposition within a genome, the closest relative of each LTR must be generally found on the same genome. Oppositely, admixed genomes showed a completely different behavior: since admixtures are recent events, most retrotransposons have not yet replicated in the admixed genome, and therefore the transferred unpaired LTRs are more closely related to those present in the original species or in other admixtures derived from these species. These results highlight the importance of admixtures in the generation of novel LTRs combinations (and potentially retrotransposons) by combining haplotypes from different origins, a hypothesis proposed in one of the earliest transposon studies (Suoniemi et al. 1998). Although most LTRs followed the abovementioned trend, some of them found their closest relatives in distant species (for instance, Clementine’s LTRs whose closest relative was detected in *S. buxifolia* or *C. ichangensis*). Even though this observation may certainly pinpoint to a failure in the detection of their closest homologues, the occurrence of closely related LTRs in highly divergent species supports the idea that they can indeed persist over long periods of time even when the retrotransposon itself is no longer present (Ma and Bennetzen 2004; Hawkins et al. 2009).

### Mechanisms of Retrotransposon Accumulation in *Citrus*

Regarding the retrotransposon distribution across the genome, we first focused on the *C.clementina* genome. The genic content per genomic window was used to roughly estimate the location of pericentromeric regions in the different chromosomes (fig. 2*d*), that was generally in accordance with previously reported centromere locations (Wu et al. 2014; Aleza et al. 2015). Pericentromeric regions were indeed enriched in LTR retrotransposons while the genic abundance was low (fig. 2*a*), a pattern conserved in all genomes analyzed (supplementary table 2, Supplementary Material online) in line with previous findings in *Citrus* (Du et al. 2018; Liu et al. 2019) and other species (Paterson et al. 2009; Xu and Du 2014). It is generally accepted that this pattern may arise to either a purifying selection against gene-disrupting retrotransposon insertions (Pereira 2004) or an increased unequal recombination (UR) rate in uncondensed regions (Tian et al. 2009), two processes that would reduce retrotransposon half-life in gene-rich regions and produce a preferential accumulation of recently inserted elements in them, as observed in figure 2*b*. However, both hypotheses are not mutually exclusive, and their combination actually might better explain the accumulation pattern observed in this work. Consequently, the patterns of retrotransposon insertion, accumulation, and purge were analyzed to determine their effects on shaping the studied genomes.

To understand whether UR has a decisive effect in the retrotransposon distribution, UR rates across each genome were estimated. Considering that the paired LTR to soloLTR conversion is unidirectional, the soloLTR to total LTR proportion was taken as a proxy of the soloLTR generation frequency (Cossu et al. 2017; Liu et al. 2019), which equals the intraelement UR rate. We found UR to be consistently more frequent in the genic regions of every genome analyzed (supplementary table 2, Supplementary Material online), in agreement with previous works in Arabidopsis (Pereira 2004), providing an explanation for the accumulation of complete LTR retrotransposons in pericentromeric regions. This hypothesis is further supported by the position of the retrotransposon activity hotspots found in mandarins (fig. 2*a* and supplementary fig. 2, Supplementary Material online), that were primarily located in genic regions, as observed for the tomato genome (Xu and Du 2014).

We also studied the rate of generation of nonsoloLTR to determine the sum of the interelement UR and IR rates, and found no significant or consistent variations between genic and nongenic regions in most of the genomes (fig. 2*c* and supplementary table 2, Supplementary Material online). This inconsistency together with the low number of nonsoloLTRs found in all genomes (only 30% of the unpaired LTR) may suggest that the combined effect of UR and IR is not determinant in the LTR accumulation patterns observed.

On the other hand, the increase in the retrotransposon purge rate (the sum of UR and IR purge) in the genic regions appears to account for the retrotransposon age distribution found in six out of the eight species analyzed (fig. 3), as has been described in Arabidopsis and tomato (Pereira 2004; Xu and Du 2014). In these genomes, old retrotransposons are preferentially accumulated in the pericentromeric regions, that show a reduced transposon deletion rate which in turn slows the transposon turnover while increasing their half-life (Tian et al. 2009; Pellicer et al. 2018). In citrons and pummelos, however, other different mechanisms must operate since the retrotransposon age distribution in genic and pericentromeric regions are very similar. In pummelos, new retrotransposons are preferentially inserted in pericentromeric regions leading to uniform age distributions along the chromosome but with a much larger number of retrotransposons in nongenic regions. Currently, there is not a general agreement on whether or not retrotransposons preferentially insert in some regions of the genome since evidences have been found for centromeric (Tsukahara et al. 2012) and euchromatic (Wei et al. 2016; Nakashima et al. 2018) preferential insertions, or even for a completely unbiased distribution (Levin and Moran 2011).

Apart from these mechanisms, the effect of purifying selection has been suggested to become relevant in gene-rich regions, where insertion has higher chances of reducing the overall fitness of the individuals favoring the selection of transposon-free alleles (Pereira 2004; Xu and Du 2014) without requiring recombination or leaving any detectable signature on the genome. In *Citrus*, the total LTR count is significantly higher in pericentromeric regions even if insertion is generally unbiased. This observation strongly suggests that purifying selection is playing an important role in shaping the retrotransposon landscape of *Citrus*, since that count, that is, the number of paired LTRs plus twice the number of unpaired LTRs (soloLTR and nonsoloLTR), is not constant across the genome (fig. 2*c*), as expected when insertion is uniformly distributed.

Although multiple studies have reported the accumulation of complete LTR retrotransposons in pericentromeric regions, here we extend this concept and propose that the total LTR count is an indicator of retrotransposon purge through mechanisms other than recombination, provided the occurrence of unbiased insertion. It is worth to mention that differences in the selective pressure could modulate the reduction of the number of young elements in the genic regions, shifting the distribution toward older ages to distinct levels. Thus, an increased selective pressure might produce, for instance, the pattern depicted for *C. medica* in figure 3. Therefore, our results suggest that the retrotransposon accumulation pattern found in the eight genomes analyzed might be explained by the combination of UR purge and purifying selection, whose combined effect permits the pericentromeric regions of *Citrus* and *Severinia* genomes to behave as safe havens for retrotransposons, as described in many plants (Pereira 2004; Levin and Moran 2011).

### Regulation of Retrotransposon Activity during *Citrus* Speciation

It is generally accepted that retrotransposon insertion rate continuously increases over time while the purge rate remains constant. Based on these premises, LTR age distribution has been suggested to follow an exponential growth curve, as modeled in multiple species including *Citrus* (Wicker and Keller 2007; Hawkins et al. 2009; Liu et al. 2019). Although retrotransposon removal is in principle an unspecific process derived from recombination, retrotransposon activity appears to be a clearer target for differential regulation. Consequently, the number of elements detected in each bin has been repeatedly used as a proxy to date retrotransposons in several works (Hu et al. 2011; Bousios et al. 2012; Zhang and Gao 2017). However, some authors suggest that the commonly observed ever-growing profile of retrotransposon activity might be indeed produced by retrotransposon removal process, that steadily deletes elements (Dai et al. 2018). This vision implies that the old elements that are detected in current genomes are those that survived by chance all this time, while the deleted elements are systematically disregarded as they are no longer present in the genome. Under these circumstances, the age distribution is not exactly comparable with the insertion history, but rather a proxy that underestimates the insertion rate values, especially in older age bins. However, as long as the deletion rate does not abruptly change among species, the age distribution shape in the most recent times should resemble that of the insertion history.

In this work, retrotransposons were independently dated in every superfamily, lineage of retrotransposons, and *Citrus* species (fig. 5). Within a given species, activity of both *Copia* and *Gypsy* superfamilies followed similar patterns, although each species developed a specific pattern of change. The results show that the species-specific patterns of transposon activity detected in the *Citrus* genomes can be basically grouped in three models: a) exponential or continuous increase over time (*C. clementina*, *C. maxima*, and *C. ichangensis*), b) initial continuous increase followed by a sudden arrest and a final phase of gradual reduction (*C. unshiu*, *C. reticulata*, and *C. medica*), and c) initial increase, sudden arrest, reduction, and a final period of regrowth (*C. sinensis* and *S. buxifolia*).

The observation that genomes from pure *Citrus* species sharing a recent common ancestor (*C. maxima* and *C. medica* diverged ∼6 Ma; Wu et al. 2018) exhibit different patterns of activity suggests that such activity may evolve independently in species with a common ancestor and therefore, that the phylogenetic relatedness of the genomes is not necessarily associated with their activity pattern. The same conclusion can be inferred from the comparison of other pure species pairs such as *C. maxima* and *C. ichangensis* (that shared their last common ancestor 8 Ma; Wu et al. 2018) since both followed the same activity pattern type a. These evidences highlight the different transposon activity profiles that can be found even in relatively close genomes, as previously suggested (Hawkins et al. 2009; Zhang and Gao 2017). In general, transposon activity among similar species tend to evolve in parallel (Kim et al. 2017) while more distant species do not present analogous activity trends (Wicker and Keller 2007; Xu and Du 2014), although this is not always the case (Estep et al. 2013).

Remarkably, the patterns of activity change in *Citrus* show two observations of relevance that are apparently connected. One is that the speed of change among the different *Citrus* species is extremely fast when compared with those published up to date in other plants (Estep et al. 2013; Piednoël et al. 2013; Xu and Du 2014; Kim et al. 2017). Moreover, in three out of the five pure species analyzed (*C. reticulata*, *C. medica*, and *S. buxifolia*) the increase of transposon abundance is strikingly arrested at similar K2p distance units (0.06–0.04). A rate of 4×10^−9^ to 5×10^−9^ silent base-pair substitution per year (De La Torre et al. 2017), multiplied by a factor of two to correct for the LTR increased substitution rate (Ma and Bennetzen 2004; Hu et al. 2011), was used to date the element insertions. These calculations revealed that the turning point dating the arrest of activity took place 7.5–4.0 Ma (using the widest intervals). Interestingly, the radiation originating the foundational *Citrus* species studied in here has been reported to occur 7.5–6.0 Ma during the Late Miocene in continental Southeast Asia (Wu et al. 2018), a period and region characterized by deep environmental changes. A causal connection of environmental changes and reprograming of retrotransposon activity would require further studies, but it is nevertheless very tempting to suggest that *Citrus* retrotransposons may also respond to the stressful conditions driving speciation, as a part of the genetic machinery responsible of adaptation. It is also worth to mention that the pattern of change of retrotransposon activity previous to the speciation processes is practically identical among all *Citrus* species analyzed (fig. 5) as theoretically expected, since these by definition come all from a common ancestor.

Furthermore, our results also suggest that the evolution of retrotransposon activity is, in principle, associated with the genealogic proximity, as observed in the three *Citrus* admixtures *C.sinensis* (sweet orange), *C. unshiu* (Satsuma mandarin), and *C. clementina (*Clementine mandarin). Actually, next generation sequencing has revealed that most important domesticated *Citrus* cultivars are in fact admixtures of true species, that are popularly recognized as oranges, mandarins, and lemons (Wang et al. 2017; Wu et al. 2018). These admixtures had distinct recent origins, but a similar genomic background composed of combinations of *C. reticulata* and *C. maxima.* Sweet oranges, that contain pummelo chloroplasts, are grouped under the binomial name of *C. sinensis*, while the term “mandarin” comprises a very heterogenic collection of genomes including pure mandarin species (*C. reticulata*) and genotypes with different proportions of pummelo introgression (i.e., *C. unshiu*, *C. clementina*, and *C. deliciosa*) in a maternal mandarin genome. Our data indicate that the genome of the Satsuma mandarin *C. unshiu*, for instance, that contains a high proportion of pure *C. reticulata* (86%,) showed resembling or parallel changes (model b) to those of the pure mandarin. Similarly, transposon activity in the orange *C. sinensis* (42% of *C. reticulata*) appears to follow a pattern (model c) intermediate between *C. maxima and C. reticulata.*

**Fig. 6.**
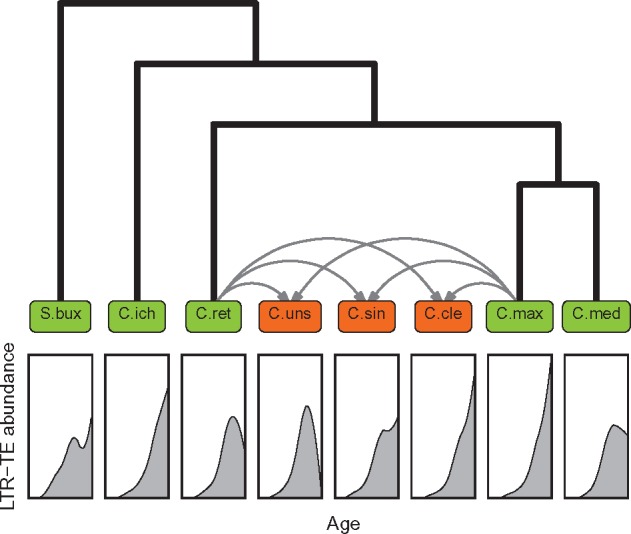
—Retrotransposon activity and *Citrus* phylogeny. Cladogram representing the phylogeny of the eight species analyzed in this study associated with the pattern of retrotransposon activity found in each one of them. Pure species are framed in green boxes while admixtures are framed in orange boxes, with gray arrows indicating their pure species progenitors. The overall retrotransposon activity evolution over time is presented below each species name. Species codes are as in figure 1.

The activity pattern (model a) of *C. clementina*, an admixture of the orange *C. sinensis* (*C. maxima*×*C. reticulata*) and the mandarin *C. deliciosa* (*C. reticulata*×*C. maxima*), was similar to that of *C. maxima* (fig. 6), although the contribution of pummelo to the Clementine genome is only of 12% (Wu et al. 2018). These observations suggest that *C. deliciosa* mandarin, whose reference genome is not available, must carry highly active retrotransposons to produce the profile observed in Clementine and that the mandarin haplotype included in *C. deliciosa* neither is the same that contains the *C. unshiu* mandarin nor is directly associated with the genome of the pure *C. reticulata* sequenced (Wang et al. 2018) and used in the current work. This last assumption is derived from this previous study (Wang et al. 2018) that divided domesticated mandarins in two different clades, one evolving through the north of the Nanling Mountains, which included *C. unshiu*, and the other expanding to the south of this mountain range and harboring *C. deliciosa*. Nanling Mountains in Southern China separate south and central subtropical zones. It is worth to mention that not only *C. unshiu* and *C. clementina* arose from different mandarin genomic backgrounds but at least four different pummelo haplotypes are also found into the genomes of these two mandarin admixtures.

Another set of interesting data come from the individualized analyses of the different retrotransposon lineages that evidences how in every species studied, some lineages did not follow the general pattern of activity of the species itself. For example, the increase in activity of SIRE elements was the highest in the past just before the beginning of the *Citrus* speciation, that is, the abundance of SIRE elements was progressively reduced in all *Citrus* analyzed, but not in *Severinia*. This together with their abundance (they rank 3rd or 4th) suggests among other possible explanations, that these elements have not been able to counteract the genomic mechanisms implicated in their silencing process in *Citrus*. On the contrary, Retrofit elements have continuously been growing over time in most of the genomes, including some of those showing different models in the general tendency, such as *C. reticulata* (model b) or *C. sinesis* (model c). Retrofit elements, therefore, show an elevated ability to overcome hosts regulation, as described previously for other lineages (Hernández-Pinzón et al. 2012; Fu et al. 2013; Lu et al. 2017). This is not a surprise since different behaviors of transposon lineages and families within a single genome have been already reported (Piegu et al. 2006; Bousios et al. 2012) and recent studies have also observed great variations on transposon activity in groups of closely related species (Estep et al. 2013; Quadrana et al. 2016; Zhang and Gao 2017; Carpentier et al. 2019).

The detailed analyses of the activity of each retrotransposon lineage revealed that only in two genomes, *C. unshiu* (model b) and *S. buxifolia* (model c), all lineages showed the same pattern. As mentioned above, *C. clementina* and *C. reticulata* followed models a and b, except for the SIRE and Retrofit families. There were two lineages that escaped to the general tendencies found in *C. sinesis* (model c), *C. medica* (model b), and *C. ichangensis* (model a). These were Tork and Retrofit in the first two genomes and Athila and Tat in the papeda. Finally, Reina, CRM, and SIRE retrotransposon families showed evolutionary trends dissimilar to the pivotal patterns of gradual growth found in *C. maxima*. Overall, these results indicate that mobile element activity in each *Citrus* genome follows a characteristic and recognizable pattern of change although very often a few retrotransposon lineages evolve independently following a different trend. Except for the SIRE elements that in *Citrus* always show a tendency of type b, all lineages show patterns that follow either models of type a or b, while many lineages of the *Gypsy* superfamily in addition exhibit models of type c.

In conclusion, our results show that in *Citrus*, retrotransposon activity in a given species or admixture is not clearly related to any fundamental genomic or phylogenetic factor. Although the pattern of activity of the *Citrus* admixtures is originally associated with the genealogic proximity of their genomes, the drastic changes in the activity that each species experiences over time appear to be mainly driven by the evolutive history of its particular genome. Interestingly, in some genomes the expected pattern of gradual transposon accumulation is strikingly arrested shortly after the radiation of the *Citrus* genus, coinciding with a geological era characterized by dramatic climate changes. Overall, our results may suggest that the retrotransposon evolutionary landscape is largely governed by the individual past of each species or population, a hypothesis compatible with the changing environmental scenarios and evolving conditions that occurred during *Citrus* speciation. Based on these observations, we propose that *Citrus* retrotransposons might respond to those stressful conditions driving speciation, as a part of the genetic machinery responsible of adaptation. This proposal implies that the evolving conditions of each species may interact with the internal regulatory mechanisms of the genome regulating proliferation of the mobile elements and that this interaction may be very subtle since it discriminates between different lineages of retrotransposons.

## Supplementary Material

Supplementary data are available at *Genome Biology and Evolution* online.

## Supplementary Material

evz246_Supplementary_Data

## Notes

**Data deposition:** The reference genomes used in the current study are available in the NCBI Assembly repository. The GeneBank assembly accession for each reference genomes are the following: *C. clementina* (GCA_000493195.1), *C. sinensis* (GCA_000317415.1), *C. unshiu* (GCA_002897195.1), *C. reticulata* (GCA_003258625.1), *C. maxima* (GCA_002006925.1), *C. medica* (GCA_002013955.2), *C. ichangensis* (GCA_002013975.2), and *S. buxifolia* (GCA_002013935.1). The Illumina-sequenced mandarin genomes are available from the Sequence Read Archive and their identifiers are provided in the supplementary table 1, Supplementary Material online. The genomic locations of every LTR retrotransposon (either complete elements or retrotransposon cores) are listed in the supplementary table 3, Supplementary Material online, based on the coordinated of the reference genomes used in this work (see Materials and Methods).
