# Supplementary figures and images for: Reprogramming of Retrotransposon Activity during Speciation of the Genus Citrus
Source: Genome Biol Evol. 2019 Nov 9;11(12):3478–95. doi: 10.1093/gbe/evz246 (PMC7145672; doi:10.1093/gbe/evz246)

Blastn

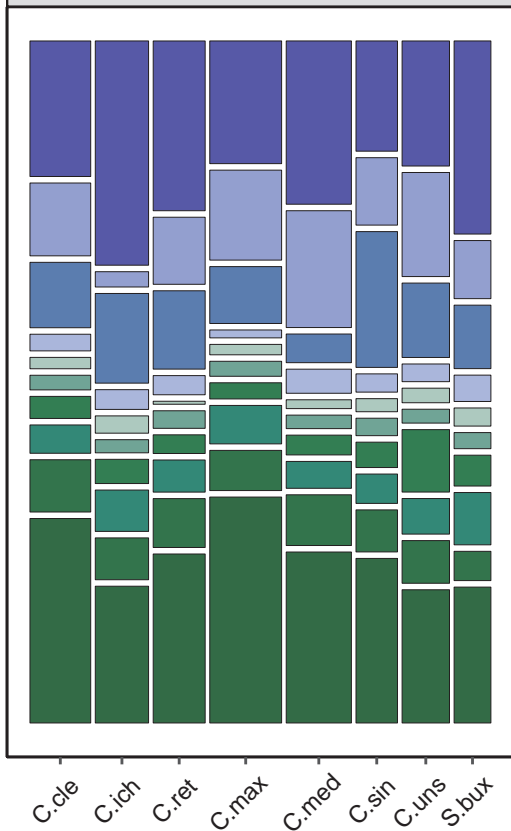

LTR\_Harvest

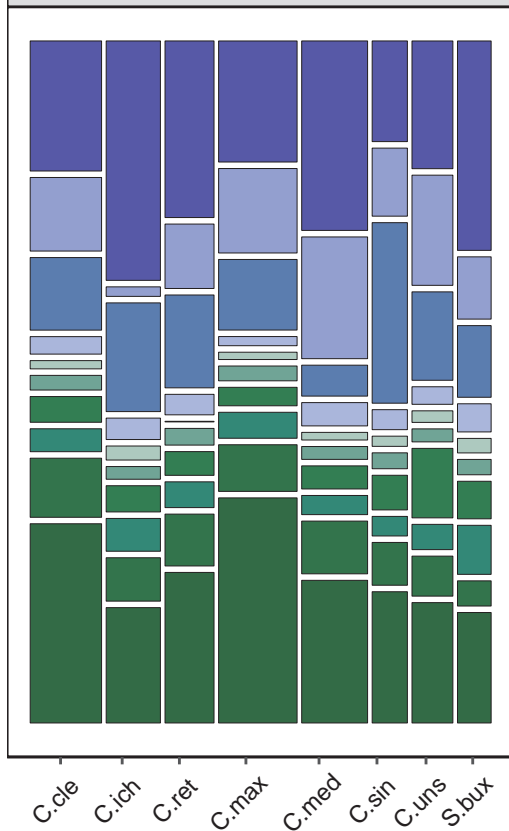

Lineage

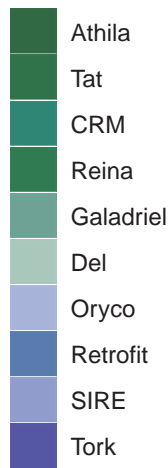

Supplement: evz246_Supplementary_Data [file evz246_supplementary_data.zip › Supplementary Figure 1.pdf]

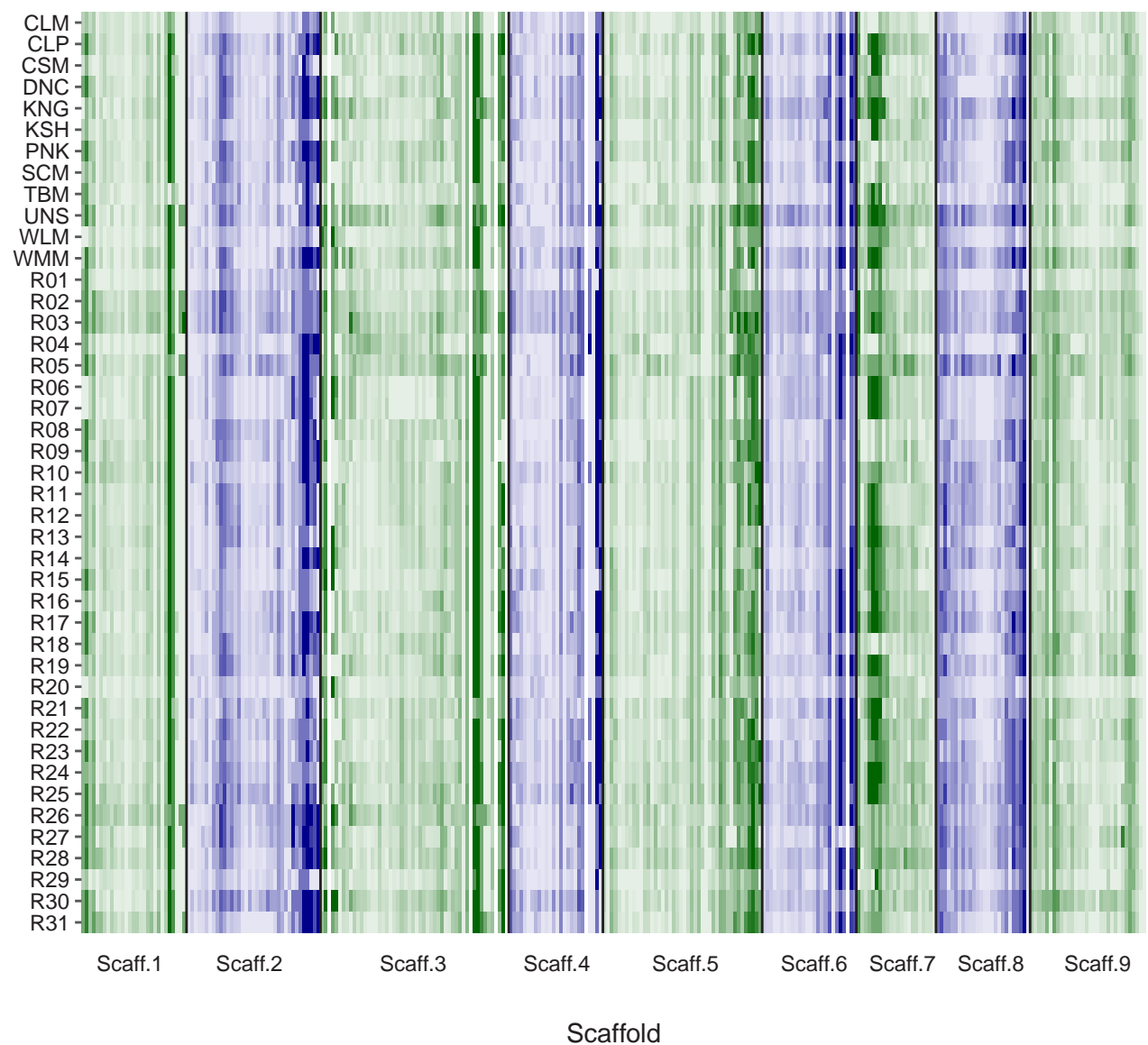

Supplement: evz246_Supplementary_Data [file evz246_supplementary_data.zip › Supplementary Figure 2.pdf]
